# Editorial: Challenges to Mean-Based Analysis in Psychology: The Contrast Between Individual People and General Science

**DOI:** 10.3389/fpsyg.2016.01234

**Published:** 2016-08-19

**Authors:** Craig P. Speelman, Marek McGann

**Affiliations:** ^1^School of Psychology and Social Science, Edith Cowan UniversityJoondalup, WA, Australia; ^2^Department of Psychology, Mary Immaculate College, University of LimerickLimerick, Ireland

**Keywords:** mean, average, variability, inference, groups, individuals

In a recent paper we (Speelman and McGann) argued that psychology's reliance on data analysis methods that are based on group averages has resulted in a science of group phenomena that may be misleading about the nature of and reasons for individual behavior. The paper highlighted a tension between a science in search of general laws on the one hand, and the individual, variable, and diverse nature of human behavior on the other. Two central traditions in psychology are challenged by this tension: (1) data is collected from a large number of people and distilled into a handful of parameters that reflect the middle of a distribution of scores and the average variation around that mid-point, and (2) theories are developed to explain the average performance of the group. The disjunction between group-based measurements and the actual psychology of individual people raises specific concerns in both research and applied professional domains of psychology. For instance, a clinician who reads in a report that Therapy A leads to a significantly greater improvement in depression than Therapy B might be tempted to adopt Therapy A in her practice. But what are the odds that Therapy A will be the best option for the next depressed client to walk in her door? What does an observation that, on average, people find it easier to identify letters presented on a screen when they are presented at the end of a word than when presented in isolation actually tell us about the specific cognitive processes occurring in specific people's activities? Are we justified in interpreting this result as reflecting something about the way every person's mind processes letters and words? To what extent should we explore the prevalence of this pattern of responding before we start making claims about cognitive mechanisms that are general to all humans?

We argued that more explicit and careful justifications are required for the common practice in psychology of extrapolating from average data to general laws, but also from general laws to explanations of individual behavior. Given the ability of humans to adapt to their environments, it would seem unlikely that everyone would develop identical cognitive processes for any given task. As a result, developing general theories about any given task, and using those theories to develop methods for clinical interventions or educational purposes would seem a risky endeavor.

This Research Topic explored this concern about the pitfalls of using the mean for the basis of psychological science. The problem is universal in its applicability to psychology, and opinion papers, reviews, and original empirical research from all areas of the discipline were invited.

A total of 16 authors contributed 9 articles to the Topic. The range of issues that the authors viewed through the lens provided is impressive. These articles follow two principal themes. The first concerns the relationship between theory and different statistical techniques, and how a more comprehensive understanding of psychology demands a more varied (and perhaps more precise) set of investigative techniques. The second theme concerns more fine-grained technicalities, and the papers here illustrate the practical significance of understanding the relationship between measures of central tendency and other characteristics of the data sets that give rise to them.

Papers in the first theme explore ways in which we can discipline our data collection to avoid the traps of logic associated with careless use of averages. Campitelli, for instance, argues that psychology typically produces imprecise theories and so tends to fit its research questions to the available statistical tools. He advocates for the development of more precise theories and describes four analytical methods that he has used to answer precise research questions and which do not require the calculation of the mean. Grice also recommends the development of theoretical models that are person-centered, rather than group based, and so do not require aggregate statistics, such as the mean, to evaluate. Such an approach is perhaps more akin to a detective gathering clues to solve a mystery, enabling investigators to gather information and test specific models based on patterns of collected evidence, rather than on the success or failure of individual observations.

McAuliffe and McGann explore one particular way to gather information about the context of behavioral measurements that may highlight variability, and enable an exploration of that variability within standard laboratory tasks. They suggest adapting Hurlburt's descriptive experience sampling method for the laboratory in order to enable interrogation of behavioral performance in terms of the details and variety of individual experiences reported by participants during a given task.

Finally in this group, Kirsner's article describes the long and convoluted process involved in predicting the locations of two related shipwrecks. He shows how aggregating many disparate pieces of information pointed to the most accurate locations for these wrecks, a process he likens to the calculation of the mean or population parameter, and so highlights a situation where multiple perspectives provide a kind of parallax that can be used to bring a single target into focus, rather than depending on multiple measurements of the same variable to average out noise.

Complementary to these explorations of alternative methodological or analytic approaches are papers that illustrate and explicate more specific technical problems with various uses of the mean. In each of these papers the relationship between the mean and other aspects of the data in question can have a substantial impact on the validity of our inferential techniques, and the kinds of conclusions we might draw.

Speelman and Muller Townsend examined the extent to which average group performance can mask the heterogeneity that exists between the members of a group. They demonstrated that a substantial proportion of participants do not demonstrate a transition from controlled to automatic performance in a standard training experiment, despite the fact that the group results suggested such a transition occurred.

Looking at linear mixed-effects models as a set of analytical methods for overcoming problems associated with the mean, Lo and Andrews examine their ability to satisfy normality assumptions without the need to resort to transformation allowing investigators to work much more closely to the raw data themselves.

Hamaker and Grasman demonstrate how decisions about the centering methods used in cluster analysis can affect the ultimate solution, and that this affects levels of a multi-level autoregressive model differently. Their work emphasizes once again the importance of careful, deliberate use of our analytical tools, and that effective statistics rely on clearly set out, and explicit theorizing. Schuurman et al. work complements this somewhat examining the effect of including multiple sources of variation into a model, specifically focusing on noise in data. Mostly associated with measurement error, they show that noise can have a substantial effect on parameter estimation in autoregressive modeling. On the basis of their simulation study, they conclude that incorporating this noise into an analysis results in more accurate estimation.

Finally, Trafimow discusses how the meaning that can be attributed to the value of a sample's standard deviation can depend on the value of the sample mean, and vice-versa. Using a newly defined “coefficient of centrality” (the reciprocal of the coefficient of variation) as a means of relating the mean and standard deviation, he recommends that researchers routinely consider standard deviations when interpreting means. While other papers perhaps illustrate more dramatic departures from currently widely used practices in psychological statistics, Trafimow's work shows how relatively modest changes in our approach can provide quite striking improvements in our understanding.

Psychology as a discipline has been facing challenges that are not simply statistically significant, but practically, and perhaps fundamentally so. In our 2013 paper we noted that much in our argument was not particularly novel to psychologists, but despite a background or low-level awareness of possible problems, as a profession we have rather stubbornly pushed on with an uncritical or unthinking use of averages in our descriptions of groups, and a suppression of variation in our interpretation of results. The papers in this collection include a range of perspectives that provide concrete examples of how to approach research design, data collection, and analysis differently. No one contribution will provide a solution to our multifarious challenges, but nor should it. Our subject matter is complex and subtle, our investigations and methodological techniques will need to be equally so.

## Author contributions

All authors listed, have made substantial, direct and intellectual contribution to the work, and approved it for publication.

### Conflict of interest statement

The authors declare that the research was conducted in the absence of any commercial or financial relationships that could be construed as a potential conflict of interest.

